# Hospital and Institutional News

**Published:** 1920-09-18

**Authors:** 


### HOSPITAL AND INSTITUTIONAL NEWS.

### THE PREVENTION OF DISEASE.

The first annual report of the Health Ministry gives some interesting, if somewhat disappointing, figures in relation to the cost of the various medical services concerned. As might be expected, these costs are mounting by an enormous extent, and the urgent question of the moment is whether the country has as yet reaped a proportionate benefit. The most striking, if not most talked of, facts dealt with arise in connection with venereal disease. In 1919 apparently about 82,000 *new* cases were reported, whereas actual treatment attendances of 1919 were 1,003,000, as compared with 488,000 in 1918! Even if we admit the extending habit of seeking advice and treatment in these conditions, it seems impossible to conclude otherwise than that venereal disease is not decreasing, but that as far as this particular period is concerned, it is definitely increasing—or, at least, that our preventive measures such as now employed are not succeeding except in a very few instances. We note that a correspondent in *The Times* estimates, from the report, that something rather less than a quarter of a million sterling is being spent on the *cure* of a *preventable* (venereal) disease. And this estimate does not include the whole of the expenses incurred by voluntary organisations. With these facts before us the conclusion is insistent that either our present methods of propaganda are not effective or else that a far greater effort towards really practical prevention must be made.

### THE HEALTH MINISTRY REPORT.

The same report amply indicates the not inconsiderable national expenditure in tuberculosis matters, but over the year concerned it is only fair to remember that the colony and after-care schemes are now, at the time of their inauguration, taking up a relatively large sum, and that a judgment of their proportionate value must be made after a period of years. In any case, the 1919 figures indicate a slight decrease in both deaths from and notifications of all forms of tuberculous disease. And this fact combines with the satisfactory welfare report in sounding a more cheerful note. In these pages we have often illustrated the success which is crowning the child-welfare movement, and certainly the total of about £500,000 spent in the twelve months will be amply repaid to the nation.

### CONDENSED MILK.

We publish also on page 619 an article reviewing a whole-hearted attempt by a French medical authority to put forward, justly, the claims of condensed milk as an infant food. Many of the points he makes are of particular interest in that at the present time a Ministry of Health Committee is sitting to consider what practical regulations under Public Health legislation are necessary in the control of this form of milk. As the Ministry's recent report states, standards must be laid down to prevent the influx to this country of products of inferior quality, and the wisdom of this procedure is fully proved from the many advantages quoted by our contributor and which can be claimed for a really first-class condensed milk supply.

### A CLINIC FOR NERVE CASES.

In an influentially signed letter in *The Times*, attention is drawn to the fact that the Tavistock Clinic for Functional Nerve Cases will be opened as soon as the premises secured are ready for use, and that a sum of £2,000 to meet initial expenses is urgently needed. It is explained that, except for the provision made by the Military Pensions Board for War Cases, the supply of psychological treatment is wholly inadequate. The Tavistock Clinic will represent an attempt to bring this treatment gratuitously or at a small cost within the reach of persons of very moderate means, as is already done in Continental countries and in the U.S.A. How great the need is will, it is hoped, be universally recognised.

### PATIENTS' PAYMENTS DAY BY DAY.

Every week we hear of new additions to the list of hospitals which are instituting payments by patients, and there must be many more which quietly take the step and say little or nothing about it for publication. The West Suffolk Hospital at Bury St. Edmunds have decided to charge in-patients 10s. weekly, while at the Norfolk and Norwich Hospital the payment asked is £2 2s. a week, but we expect that this rather high rate will not be within the means of the majority. At Guy's Hospital it is asserted by the editor of *The Gazette* that the patients of the London Hospital are in many cases crossing London Bridge to escape the weekly guinea. Guy's has done nothing yet in the matter of a charge, and the hospital has always ploughed a lonely furrow in the matter of payments by soldiers or civilians. There are, however, contributing patients' wards at the great Borough hospital.

### MEDICAL TREATMENT ON HALF-PAY.

A recently published Army Council Instruction will come as a boon to officers on half-pay. In future they will receive medical attendance and hospital treatment as though they were officers on full pay, and, as a contemporary points out, the concession is welcome on three grounds. First, it will help those concerned to meet in a vital matter the increased cost of living; secondly, they will be assured of first-class medical care; and, thirdly, in a number of instances it will give them a more than deserved chance to recover some measure of war-damaged health. Only those whose disability is directly due to war stress are entitled to this relief, and half-pay officers concerned are advised, not first to seek medical advice in private channels, but to apply immediately to the G.O.C. the command in which they live

### THE MEDICAL DEFENCE UNION.

The annual report of this valuable medical society discloses steady progress and ever-increasing stability. The membership has reached a record figure, and the reserve funds are also larger than at any previous time. To meet the rising scale of law charges and the increase of salaries and expenses of all kinds, and also to extend the scope of the protection offered to the members, it has been decided during the present year to increase the subscription rate to £1 per annum. Even at this amount the premium remains astonishingly small, considering the immense load of care and of pecuniary anxiety which membership confers upon the individual. The present report discloses something of the risks to which medical practice exposes its exponents, as well as the prompt and effective relief which the Union offers. We learn that Sir John Tweedy, who has been President for the last five or six years, is about to retire from this position, which he has held with conspicuous energy and success. Few members of the medical profession have done as much or as valuable public work, in many positions, for the benefit of their fellow-practitioners and of the public as has this much-respected veteran: his successor, whoever he may be, has a splendid tradition to inherit and to live up to.

### NEWCASTLE ORTHOPÆDIC HOSPITAL OPENED.

The new orthopædic hospital at Newcastle-on-Tyne, which is an extension of the Royal Victoria Infirmary, was opened on the 9th instant by the Duke of Northumberland. There are 500 patients in the institution and 1,200 out-patients. When the wounded and disabled have been treated those injured in the big industries of the locality will be admitted. The buildings occupy fifteen acres, and have cost £160,000, towards which £148,000 has been subscribed. The medical superintendent is Lieut.-Col. F. E. Bissell.

### A HOSPITAL CRICKET DAY.

The suggestion has been made that cricketers do less than football players for hospitals, and the M.C.C., it is proposed, should use its influence to promote a Hospital Cricket Saturday, when the proceeds of all the matches throughout the kingdom should be given to the hospitals. We hope that the suggestion will be considered by all the cricket clubs, and that the M.C.C. will encourage the proposal. There is plenty of time to arrange matters between the end of the present and the beginning of next year's season, and an enthusiast for the proposal in Oxford urges that, since footballers have been the first in the field, they might extend their help and be the first to start a Hospital Football Saturday also. The writer urges this upon the Oxford Football Clubs, partly because they already help hospitals and their season is about to begin, and partly because their example would prepare the way for any proposals that the M.C.C. may be ready to consider. We hope that the idea may not be allowed to drop, and would welcome the views of the authorities at Lord's upon the matter.

### THE GREAT NORTHERN EXTENSION.

A Flag Day in aid of the extension of the Great Northern Hospital is to be held in Islington on Wednesday next, September 15.

### LINCOLN RED CROSS CONFERENCE.

The first of a series of conferences between county representatives of the Order of St. John of Jerusalem and the British Red Cross has been held at Lincoln. Fifty delegates from Lincolnshire, Nottinghamshire, Derbyshire, and Leicestershire responded to the invitation of Colonel Walker, County Director for Lincoln, to hear speeches by Sir Arthur Stanley and Sir Napier Burnett. The object of the conferences is to set up joint county committees of the two Societies to organise voluntary effort for the sick. Lord Monson, St. John Commissioner in Italy, presided. Sir Arthur Stanley said that the work could only be maintained by county organisations, and counties liked to manage matters in their own way. There was an immense amount of work to be done. The Joint Committee received daily demands from hospitals for exactly the same luxuries and medical stores with which they used to supply military hospitals. He then went over the programme of objects in view—welfare work, sanatoria, motor ambulances. Money was needed for it. Hence the decision to hold an " Our Day " collection, which, he observed, had not been rapturously received. Of the money collected in the counties 75 per cent. would be spent locally and 25 per cent. would go to headquarters to maintain the central organisation. Sir Napier Burnett called attention to the prospect of a serious shortage of nurses, and again drew attention to the financial needs of the provincial hospitals, which, he said, required one million to be restored to their pre-war position. It is hoped that these conferences will also stimulate public interest and lead to thorough preparation for Red Cross Week, from the 11th to the 17th of next month.

### LONDON RED CROSS CONFERENCE.

To help the Red Cross Week, which will be held from October 9 to 17, county conferences to discuss local plans are being arranged. The London Conference will be held on Monday, September 13, at 27 Grosvenor Place, at 3 p.m., the office of the London County Director, at which the counties of London, Middlesex, Essex, Kent, Surrey, Sussex, Bucks, Herts, Berkshire, and Oxfordshire will be represented. The Birmingham Conference will be held on September 16 in the Council Chambers at 12 noon, and the Manchester Conference on September 17.

### HONOUR FOR THE SECRETARY OF THE ITALIAN HOSPITAL.

The Cross of Chevalier of the Order of the Crown of Italy has been conferred by the King of Italy on Mr. Frederick Hornyik, secretary and controller of the Italian Hospital, London.

### THE RED CROSS PROPAGANDA.

BY the time we go to press the first meetings of the Country Campaign, undertaken by Sir Arthur Stanley and Sir Napier Burnett will have been held. In the meantime the help of a number of public persons has been enlisted and they have issued for publication an expression of their sentiments with regard to the work that has been undertaken by the Joint Council of the Order of St. John and the British Red Cross Society. It is not quite clear even now if the scheme that the Joint Council are entering upon includes the hospitals of London as well as of the provinces, but the sum which the public is asked to subscribe in connection with the movement is in round figures £1,000,000. As Sir Napier Burnett estimates the cost of arrears in the matter of buildings, alterations and cleaning at £750,000, the million appears to be rather on the moderate side. He thinks the money will be forthcoming and is of opinion that Great Britain, even in these days when economy is the highest form of virtue, is not going to tighten her purse-strings when the welfare of a suffering population is at stake.

### SOME OF THE PROPAGANDISTS.

AMONGST those who are lending their aid as propagandists are Mrs. Henry Fawcett, the well-known publicist and writer on economics; Mrs. J. R. Clynes, the wife of the famous Labour leader; Mr. Ben C. Spoor, M.P., and Mr. William Graham, LL.B., M.P. The latter, who sits for the South Edinburgh division, has the distinction of being the only University graduate in the Labour party at present in the House of Commons. He is recognised as one of the most level-headed of his party with whom its future will probably be largely associated. It is very appropriate that the representatives of Labour should be connected with the Red Cross scheme, as no subject is of such paramount importance to the working classes as the proper development of our National Health services. When the wage-earner of the family is stricken by illness, defects and benefits of medical systems are brought right home. The working people of the country have responded most liberally to the increased needs of the hospitals during the past few years and their organisations, especially in the provinces, for contributing to hospitals are excellent. In making the appeal for the round million, the point is emphasised that this will not bring the institutions out of their difficulty; the sum will simply place them in the same position they were in before the war, but it is clear that a much larger annual income will be necessary to enable them to meet their increased running expenses quite apart from any sum needed for increasing their efficiency. However, a scheme such as that which has been embarked upon must have the effect of winning to the cause of the hospitals many people who hitherto have never given material assistance, and of them no doubt a large proportion will remain permanent friends and subscribers.

### PAYMENTS BY DENTAL PATIENTS.

THE Committee of the Royal Dental Hospital, Leicester Square, W.C., has issued a notice to the subscribers and life governors stating that it has been found necessary to make a small charge to patients attending the hospital. This step is not surprising when one considers the very expensive nature of the services given to dental patients, and the large cost of upkeep of equipment necessary in such an institution as the Royal Dental Hospital. The Committee thinks there is little doubt that the patients are drawn largely from those who derive benefit from the present high rate of wages, while the voluntary revenue received by the hospital comes from others whose incomes are seriously affected by increased taxation. The Committee, therefore, feel it their duty to obtain a small fee from patients before making a further appeal to an already overburdened charitable public. We have no doubt that the patients will appreciate the justice of this new arrangement and will respond as cheerfully as they have done in other hospitals which have found it necessary to request the patients to help towards the cost of their treatment.

### TUBERCULOSIS ARRANGEMENTS PROTEST.

AT a meeting at Worcester of the County Insurance Committee, some concern was manifested over the future administration of sanatorium benefit (of course, it will be remembered that shortly, in all probability, such administration will be removed from the hands of Insurance Committees to those of the local sanitary authorities). Mr. Willis Bund, who is chairman of both the Worcestershire Insurance Committee and of the Worcestershire County Council, stated that he had recently written to the Ministry of Health stating that unless the Council received a very substantial grant from the Treasury, it would be compelled to cut down very greatly the sum now expended in sanatorium treatment or else to decline to receive tuberculosis patients. The county scheme last year cost £13,000, of which the rates provided only £4,500. It had been stated that the Ministry intended to make County Councils an annual grant of 50 per cent. in respect of expenditure on sanatoria. Such expenditure, however, would obviously increase the running expenses. The Ministry of Health, in reply, stated that the matter was under consideration, and that no decision had yet been arrived at. Communications would be addressed to County Councils and to Insurance Committees in due course explaining the arrangements to be made for the institutional treatment of tuberculosis on and after January next. Tuberculosis workers, therefore, must meanwhile possess their souls in patience.

### FUTURE OF OTLEY INFIRMARY.

AT the laying of the foundation-stone of a new operating-theatre at the New Hall Institution, Otley, Yorkshire, some interesting speeches were made. Dr. Hebblethwaite, chairman of the house committee, said that the new theatre would bring the surgical equipment of the infirmary up to the modern standard, and that whatever happened to the Guardians, the institution would be in excellent order when the time came for them to retire. Perhaps it would be the nucleus of a big Wharfedale Hospital, wholly independent of the Poor Law, and, since the nearest general hospitals were at Bradford and Leeds, Otley Infirmary might be a half-way house between the free general hospitals of the big towns and the expensive nursing homes.

### PATROL OFFICERS FOR SANATORIA.

Indiscipline among sanatoria patients is a recurring complaint, perhaps because too many medical superintendents have been appointed of recent years without special experience either of the disease or of the management of this particular class of patient. At Winsley Sanatorium, where Dr. C. F. Pedley, senior resident medical officer, has lately succeeded Dr. Melville Rees, the chairman of the executive, Mr. Henry Anstey, records two causes of complaint. The first concerns the increasing number of visitors, particularly on Sundays, some of whom have been damaging trees and flowers and indulging in unseemly behaviour. The governors wish, he said, to give every encouragement for visits from the families or friends of patients, but could not suffer conduct of this kind. He had also received complaints of some of the patients when outside the grounds of the sanatorium. A patrol officer for the precincts had been suggested, but Mr. Anstey hoped that such a plan would prove unnecessary. It should certainly do so, since the patients themselves have a personal interest in the preservation of grounds which their own work has helped to beautify.

### PORTSMOUTH PATIENTS' PAYMENTS CRITICISED.

The decision a month ago to impose a charge of 15s. a week for adult and 7s. a week for child patients at the Royal Portsmouth Hospital has not been free from criticism. Presenting the report of the Admissions Committee, Mr. J. Smyth, its chairman, said that some of the patients had been subjected to a cross-examination of which he could not approve. Against his advice, some of the committee had compelled patients to give more than they said they were able to give. Another speaker also declared that she was much opposed to the way in which some of the patients had been spoken to. Dr. C. P. Childe, who addresses meetings of workers, said that a payment of 2d. a week would have been a much better way of raising funds. The scheme is to have a trial for six months, and Mr. T. H. F. Lapthorn urged that this trial should be carried out. The criticisms are remarkable, because they come from the chairman of the Admissions Committee and because the vice-chairman, Miss Reed, declared, in an attempt to palliate them, that she did not think the treatment of patients had been more severe than hitherto! Such a defence is very dubious, and when we recall the want of support of which the hospital has complained in the past we wonder whether the Admissions Committee has been the cause of that indifference. Such a committee can easily gain a hospital a bad reputation, whereas its aim should be to make a firm friend of every applicant who comes before it.

### A HOSPITAL'S FIRST LEGACY.

A legacy of £300 has been bequeathed by Miss Garland, of Retford, to the Alexandra Hospital, Woodhall Spa, Lincolnshire. This is the first legacy ever bequeathed to the hospital, which was founded in 1890.

### SECTIONAL SUBSCRIPTIONS AT COVENTRY.

Mr. E. Victor Dodd has been able to report good progress with the scheme for raising subscriptions from each class to the Coventry and Warwickshire Hospital. A number of firms, he says, has responded to the appeal to employers, and further help from them is expected. The professional classes have been approached in their turn, and bank clerks and accountant's clerks have promised to help. The city police have agreed to contribute threepence per week from each member of the force. We hope that retailers will not be forgotten. Every business which comes under the Factory and Shops Act is eligible for a fund, and if this is raised on the basis of a weekly contribution with the head giving a proportionate sum much will be done to retrieve the financial position. We live in a time when large sums, like large profits, are mainly raised by innumerable shillings and pence. Voluntary finance needs to adopt this method of income, and it should be applied to every class which a hospital serves. We congratulate Mr. Dodd and his committee on the success which has attended the state of the new plan at Coventry. Every such example strengthens the voluntary system at the present time.

### WELCOME GIFT FOR NORTHAMPTON HOSPITAL

The United Services Fund has promised £2,500 or £3,000 to the Margaret Spencer Home of Rest, in connection with Northampton General Hospital, on condition that the Fund shall be allowed two representatives on the board of management. The offer has been accepted with gratitude. The restoration fund is now in need, for an extensive list of alterations is planned, including a new nurses' home and the rebuilding of the kitchens.

### THIS WEEK'S DRUG MARKET.

Buying continues to be confined to small purchases sufficient to cover immediate requirements, and most of the price changes are again in a downward direction. A considerable reduction in the value of cocaine hydrochloride is to be noted, but the demand is small at the moment. Opium is unchanged in price, but it is difficult to foresee how the restrictions will affect values. The high price of olive oil is firmly maintained. Eucalyptus oil continues to attract attention. Phenacetin, phenazone, aspirin, sulphonal, barbitone and other synthetic drugs continue very quiet with prices barely maintained. There is no change in the position of potassium bromide, which is in plentiful supply. English refiners of camphor have reduced their quotations. Citric and tartaric acids are very quiet and cheaper.

